# Revealing the different performance of Li_4_SiO_4_ and Ca_2_SiO_4_ for CO_2_ adsorption by density functional theory†

**DOI:** 10.1039/d2ra01021f

**Published:** 2022-04-11

**Authors:** Wenjing Yu, Qian Xu, Shenggang Li, Xiaolu Xiong, Hongwei Cheng, Xingli Zou, Xionggang Lu

**Affiliations:** Shanghai University China; Shanghai Advanced Research Institute China

## Abstract

To reveal the difference between Li_4_SiO_4_ and Ca_2_SiO_4_ in CO_2_ adsorption performance, the CO_2_ adsorption on Li_4_SiO_4_ (010) and Ca_2_SiO_4_ (100) surfaces was investigated using density functional theory (DFT) calculations. The results indicate that the bent configuration of the adsorbed CO_2_ molecule parallel to the surface is the most thermodynamically favorable for both Li_4_SiO_4_ and Ca_2_SiO_4_ surfaces. The Li_4_SiO_4_ (010) surface has greater CO_2_ adsorption energy (*E*_ads_ = −2.97 eV) than the Ca_2_SiO_4_ (100) surface (*E*_ads_ = −0.31 eV). A stronger covalent bond between the C atom of adsorbed CO_2_ and an O_S_ atom on the Li_4_SiO_4_ (010) surface is formed, accompanied by more charge transfer from the surface to CO_2_. Moreover, the Mulliken charge of O_S_ atoms on the Li_4_SiO_4_ (010) surface is more negative, and its p-band center is closer to the *E*_f_, indicating O_S_ atoms on Li_4_SiO_4_ (010) are more active and prone to suffering electrophilic attack compared with the Ca_2_SiO_4_ (100) surface.

## Abstract 2

The Li_4_SiO_4_ (010) exhibits greater adsorption towards CO_2_ than the Ca_2_SiO_4_ (100) with a stronger covalent bond and more charge transfer between the surface and CO_2_.

## Graphical abstract

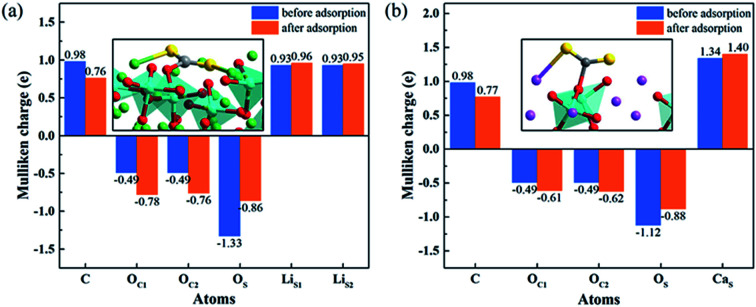

## 1. Introduction

**Fig. 1:**
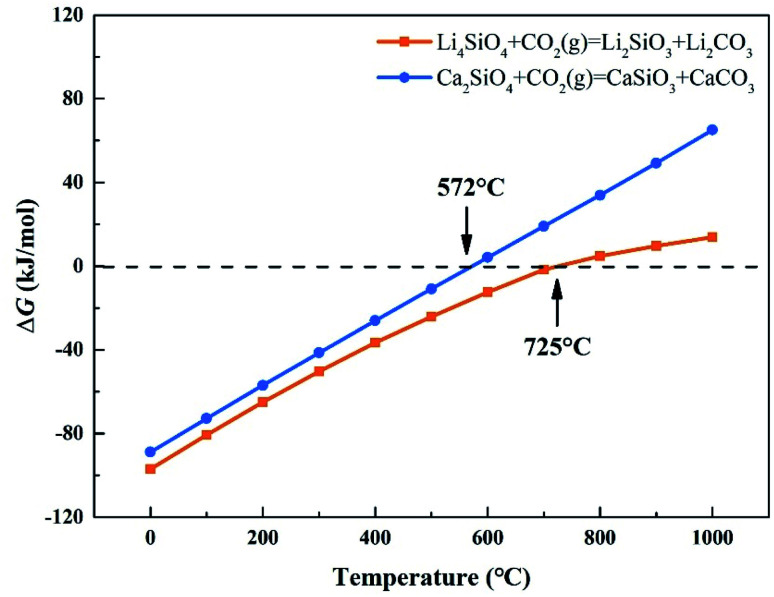
Relationship between Δ*G* and temperature of carbonation reaction between CO_2_ and Li_4_SiO_4_ and Ca_2_SiO_4_.

CO_2_ capture, storage and utilization (CCSU) is considered as one of the most promising technologies for reducing anthropogenic CO_2_ emission, which can lead to global warming. Solid inorganic sorbents have been proven to efficiently remove CO_2_ at high temperatures, and are more economical and effective than low-temperature amine-based materials in CO_2_ capture from high temperature exhaust gas.^1^ Lithium orthosilicate (Li_4_SiO_4_) is one of the best CO_2_ capture sorbents due to its significant advantages, such as large adsorption capacity, low regeneration temperature, and good adsorption and desorption cycle stability.^2–4^ There have been a lot of experimental studies on Li_4_SiO_4_ as an adsorbent to capture CO_2_, including the synthesis method,^5,6^ kinetic behavior^7–9^ and modification of Li_4_SiO_4_.^10–12^ However, lithium is relatively expensive and not very abundant in the earth's crust. In particular, lithium batteries have been widely used as a source of power or energy for a lot of things from portable electronics to electric vehicles. As a result, the demand for lithium is increased which leads directly to an increase of its price. Accordingly, it is very difficult to apply lithium-based ceramics on a huge scale to capture CO_2_ economically and sustainably. Meanwhile there are abundant resources basic silicates all over the world, especially calcium silicates (Ca_2_SiO_4_) are often found in the industrial by-products named as slags generated during iron and steel production. Furthermore, Ca_2_SiO_4_, similar to Li_4_SiO_4_, is thermodynamically favorable for CO_2_ capture from room temperature to 572 °C at ambient pressure, and Gibbs free energy changes for the carbonation of Ca_2_SiO_4_ and Li_4_SiO_4_ were calculated by HSC Chemistry 6.0 and shown in Fig. 1. However, the slow diffusion and reaction of carbonation between CO_2_ and calcium silicates is a common issue even at high temperatures in the case of no participation of water.^13^ It was found that the amount of CO_2_ captured with Ca_2_SiO_4_ is little at the temperature range from room temperature to 572 °C in our previous study. Zhao *et al.*^14,15^ applied Ca_2_SiO_4_ as the inert material to enhance the sintering resistance and cyclic stability of CaO during multiple sorption/desorption of CO_2_. It could be deduced that Ca_2_SiO_4_ is much inerter for carbonation compared with Li_4_SiO_4_ at the high temperatures.

The investigation on the effect of the electronic structure of the silicates on their carbonation reactivity should be very important for understanding deeply the different carbonation behaviors for Li_4_SiO_4_ and Ca_2_SiO_4_, and developing new approaches to improve the carbonation activity of silicates.

There are some investigations about CO_2_ adsorption on the surface of the oxides and silicates with the first-principles calculations. Kim *et al.*^16^ made an assessment of Li_2_O and Na_2_O surfaces for CO_2_ adsorption based on DFT calculations. They found that the introduction of dopant atoms larger than host metal atoms of the surfaces can negatively increase CO_2_ adsorption energies. Kumar *et al.*^17^ studied the CO_2_ adsorption on different terminations of Cr_2_O_3_ surfaces with DFT calculations and found that carboxylate species are formed on O layer terminated-(0001), and carbonate species are formed on O layer terminated-(101̄2) and Cr layer terminated-(011̄2), indicating that the formation of physisorbed and chemisorbed species depends on different surface terminations. Kang *et al.*^18^ thermodynamically evaluated the CO_2_ capture potential of Mg_2_MO_4_ (M = Si, V, and Ge). Their results indicated that the critical temperature at which CO_2_ can be absorbed, increased with decreasing Pauling electronegativity of the M site.

There are several investigations for Li_4_SiO_4_ on its structural, electronic, lattice dynamical and thermodynamic properties. Duan^19^ and Tang *et al.*^20^ found the covalency properties of Li_4_SiO_4_ mainly resulting from the overlap of O 2p and Si 3p orbitals. Kong *et al.*^21^ studied the adsorption mechanism of H_2_O on the Li_4_SiO_4_ (010) surface. It was suggested an interaction between adsorbed H_2_O and Li_4_SiO_4_ (010) surface, including an electrophilic interaction of hydrogen atom in water with oxygen atoms on the surface and a nucleophilic interaction of oxygen atoms in water with Li atoms on the surface.

Ca_2_SiO_4_, as the industrial cement clinkers, has been investigated by DFT calculations extensively, and many studies focused on the hydration of Ca_2_SiO_4_ phases. Qi *et al.*^22^ investigated H_2_O adsorption on low-index surfaces of Ca_2_SiO_4_, indicating that electron are mainly transferred from surface atoms to H_2_O molecule. Wang *et al.*^23^ evaluated H_2_O adsorption on β-Ca_2_SiO_4_ surfaces and found a dual interaction between H_2_O and β-Ca_2_SiO_4_ (100) surface. Wang *et al.*^24^ also studied the relationship between reactivity and electronic structure of 
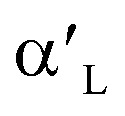
-, β- and γ-Ca_2_SiO_4_ for hydration process. They found that the higher hydration reactivity of 
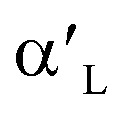
- and β-Ca_2_SiO_4_ compared with γ-Ca_2_SiO_4_ are attributed to the higher charge density and larger local state density of the active oxygen atoms in 
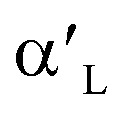
- and β-Ca_2_SiO_4_. However, there are few investigations about explanation of the different behaviors of carbonation of Ca_2_SiO_4_ and Li_4_SiO_4_ on the base of their structural and electronic properties.

Herein, we have systematically investigated the structural and electronic properties of Li_4_SiO_4_ and Ca_2_SiO_4_, and the adsorption of CO_2_ on the most stable surfaces of Li_4_SiO_4_ and Ca_2_SiO_4_ on the base of density functional theory calculations. We tried to reveal the relationship between the electronic structures of Li_4_SiO_4_ and Ca_2_SiO_4_ and their reactivity for CO_2_ adsorption on the molecular scale. The results of this investigation could converge to a proposed mechanism of CO_2_ capture with the orthosilicates, on which the more reactive silicates for CO_2_ capture can be screened out as the candidates for CO_2_ capture.

## 2. Computational details

The calculations were performed based on density functional theory (DFT), using Cambridge Series Total Energy Package (CASTEP) code.^25^ The exchange-correlation potential was approximated within the generalized gradient approximation (GGA)^26^ using the Perdew–Burke–Ernzerhof (PBE) functional.^27^ Dispersion-corrected calculations^28^ were performed with Grimme's DFT-D3 methodology.^29^ To model Li_4_SiO_4_ and Ca_2_SiO_4_, the unit cell (1 × 1 × 1) was applied for the calculation.

In order to optimize the crystal structures, the plane wave truncation energy and k-points were tested. A cutoff-energy of 650 eV was used for plane wave expansions. The k-points meshes within Monkhorst-Pack^30^ framework were set as 3 × 6 × 2 and 4 × 3 × 2 for Li_4_SiO_4_ and Ca_2_SiO_4_ respectively. The Broyden–Fletcher–Goldfarb–Shenno (BFGS)^31^ minimization algorithm was used to optimize the primitive unit cell. The surfaces of Li_4_SiO_4_ and Ca_2_SiO_4_ were cleaved from the optimized bulk structure. All surfaces were kept stoichiometric and neutral to avoid the polarizing electric field. The thicknesses of vacuum layer were set as 15 Å to avoid the interaction between slabs. The convergence criteria were fixed, specifically: the energy change within 1 × 10^−5^ eV per atom, the force on the atoms within 0.03 eV Å^−1^, the stress on the atoms within 0.05 GPa, and the displacement of atoms within 1 × 10^−3^ Å. All the initial crystal structures and date were obtained from the Inorganic Crystal Structure Database (ICSD).^32^

## 3. Results and discussion

### 3.1. Structural and electronic properties of bulks

**Fig. 2:**
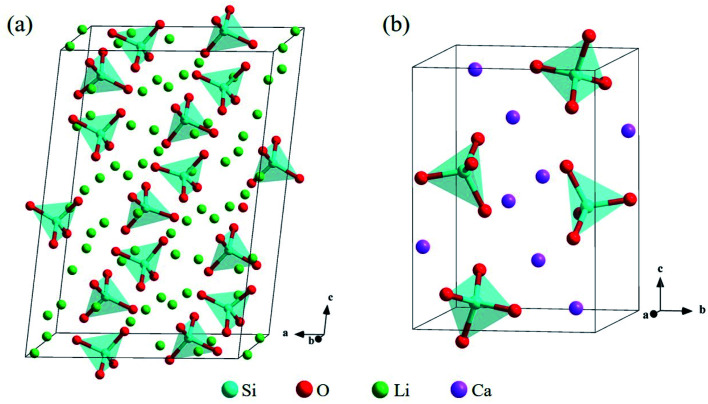
Crystal structures of (a) Li_4_SiO_4_ and (b) Ca_2_SiO_4_. The Si, O, Li and Ca atoms are shown by blue, red, green and purple spheres, respectively.

The bulk structure of Li_4_SiO_4_ with the monoclinic phase, which space group is *P*21/*m* (no. 11),^33–35^ was optimized. The unit cell of Li_4_SiO_4_ contains 126 atoms, including 14 [SiO_4_]^4−^ tetrahedra and 56 Li atoms, as shown in Fig. 2a, which are centrally symmetrical. Meanwhile, the bulk structure of Ca_2_SiO_4_ with the monoclinic phase, which space group is *P*21/*n* (no. 14),^36–38^ was optimized as well. The unit cell of Ca_2_SiO_4_ consists of 28 atoms, including 4 [SiO_4_]^4−^ tetrahedra and 8 Ca atoms, as shown in Fig. 2b. The calculated and experimentally measured lattice parameters of Li_4_SiO_4_ and Ca_2_SiO_4_ are presented in Table 1, and they are in good agreement, implying the simulation settings are reliable and give reasonable results.

**Table 1:** Comparison of calculated lattice constants of Li_4_SiO_4_ and Ca_2_SiO_4_ with experimental lattice constants

	Li_4_SiO_4_			Ca_2_SiO_4_		
	Cal	Expt^19^	Δ (%)	Cal	Expt^40^	Δ (%)
*a* (Å)	11.511	11.532	0.18	5.571	5.502	1.25
*b* (Å)	6.080	6.075	0.08	6.800	6.745	0.82
*c* (Å)	16.708	16.678	0.18	9.354	9.297	0.61
*β* (°)	99.15	99.04	0.11	94.295	94.590	0.31

**Fig. 3:**
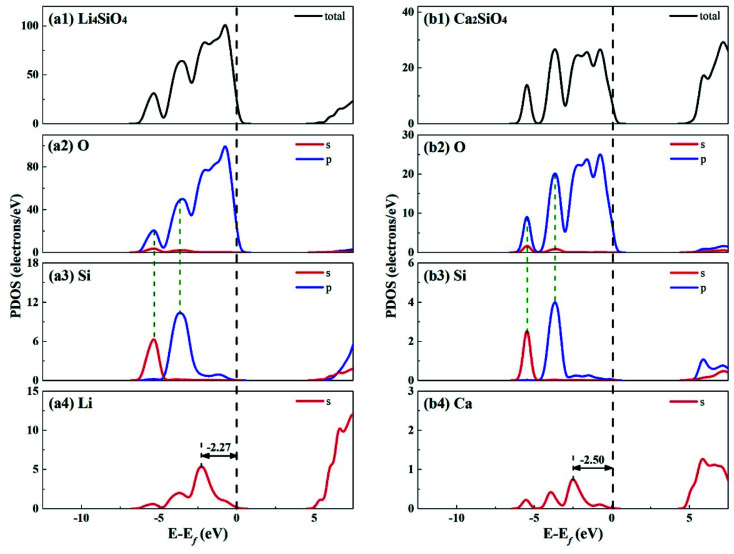
DOS analysis for (a) Li_4_SiO_4_ and (b) Ca_2_SiO_4_. The black dashed line shows the Fermi level.

Fig. 3 shows the total density of states (TDOS) and partial density of states (PDOS) for Li_4_SiO_4_ and Ca_2_SiO_4_. Electrons occupying the orbitals below and near the Fermi level (*E*_f_) is of great significance to the activity of the crystal materials for chemical reactions,^39^ so we focused on the electrons on the orbitals below and near the *E*_f_. For Li_4_SiO_4_ and Ca_2_SiO_4_, their TDOS near the *E*_f_ is mainly contributed by the p orbitals of O atoms, suggesting that O atoms are more active and more likely serve as the electron donors. Their PDOS in the region between −6.55 ∼ −2.92 eV and −6.25 ∼ −3.02 eV are overlapped with the Si s and p bands, implying orbital hybridization and Si–O binding in the bulks of Li_4_SiO_4_ and Ca_2_SiO_4_. However, the states in the region between −3 and 0 eV, near to *E*_f_ is about 73% of the total PDOS for O p orbitals for Li_4_SiO_4_, while that for Ca_2_SiO_4_ is about 70%. It can be deduced that there are more electronic states for O p orbitals below and near to *E*_f_ in Li_4_SiO_4_ than those in Ca_2_SiO_4,_ Then the electron transfer from O atoms occurs more easier in Li_4_SiO_4_ than in Ca_2_SiO_4_.

Furthermore, the first high peak position^41^ in the PDOS for Li s orbital of Li_4_SiO_4_, is closer to the *E*_f_ compared with that for Ca s orbital of Ca_2_SiO_4_, implying that the outer electron of Li atoms in Li_4_SiO_4_ can be transferred away easier than Ca atoms in Ca_2_SiO_4_.

**Fig. 4:**
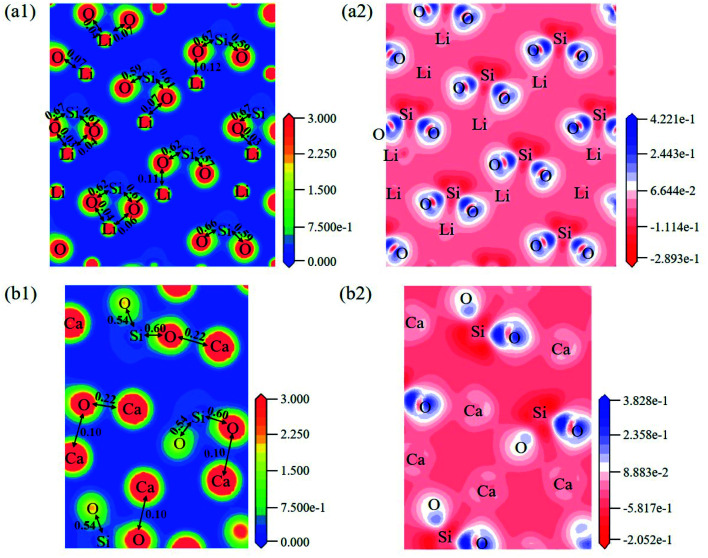
Contour maps of electron density distributions and differential charge density of (a) Li_4_SiO_4_ in the plane (010) and (b) Ca_2_SiO_4_ in the plane (100).

The electron density distribution can show the bonding between atoms and differential charge density can show the accumulation and depletion of electrons. In Fig. 4a, electron density between Si and O atoms is higher than surrounding and electrons accumulate in the middle of Si and O atoms, demonstrating that a covalent interaction of Si–O. And according to the charge population marked in the figure, the Si–O covalent interaction is stronger in Li_4_SiO_4_. Li/Ca–O have a certain covalent interaction and the strength of interaction of Li–O is weaker than Ca–O.

### 3.2. Structural and electronic properties of surfaces

In order to find out the most stable surface, the surface energies of seven low Miller index surfaces were calculated. The surface energy (*E*_surf_) can be calculated according to the eqn (1):^42,43^

**Fig. 5:**
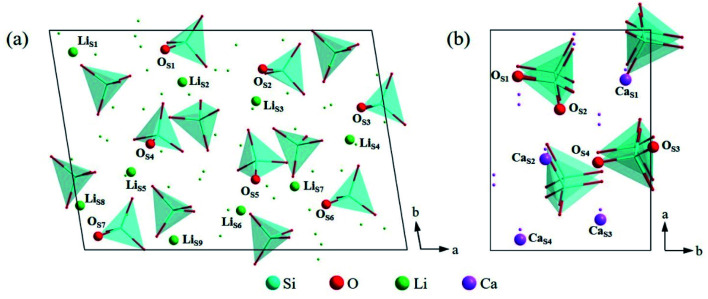
Atomic arrangement of (a) Li_4_SiO_4_ (010) surface and (b) Ca_2_SiO_4_ (100) surface from the top view. The lines and dots represent the underlying atoms and tetrahedra respectively.

1 *E*_surf_ = (*E*_slab_ − *nE*_bulk_)/2*A*

where *E*_slab_ and *E*_bulk_ are the total energy of relaxed slab model and unit cell, respectively. *n* is the number of formula units contained in the slab. *A* is the area of the slab. According to the calculation results listed in Table 2, the (010) surface and (100) surface were the most stable surface of Li_4_SiO_4_ and Ca_2_SiO_4_ respectively, due to their lowest values of surface energy, which is consistent with the previous calculations.^21,22,42^Fig. 5 shows the atomic arrangement of these two surfaces from the top view. The topmost surface layer consists of Li and O atoms for Li_4_SiO_4_, and Ca and O atoms for Ca_2_SiO_4_. The atoms of Li, Ca, and O on the topmost surface layer are referred to as Li_S_, Ca_S_ and O_S_ hereafter, respectively.

**Table 2:** The surface energies (*E*_surf_ in J m^−2^) of low Miller index surfaces of Li_4_SiO_4_ and Ca_2_SiO_4_

Surface	(100)	(010)	(001)	(110)	(101)	(011)	(111)
Li_4_SiO_4_	1.28	0.78	1.34	0.80	1.28	0.87	0.84
Ca_2_SiO_4_	0.63	1.19	0.80	0.86	0.66	0.75	0.82

**Fig. 6:**
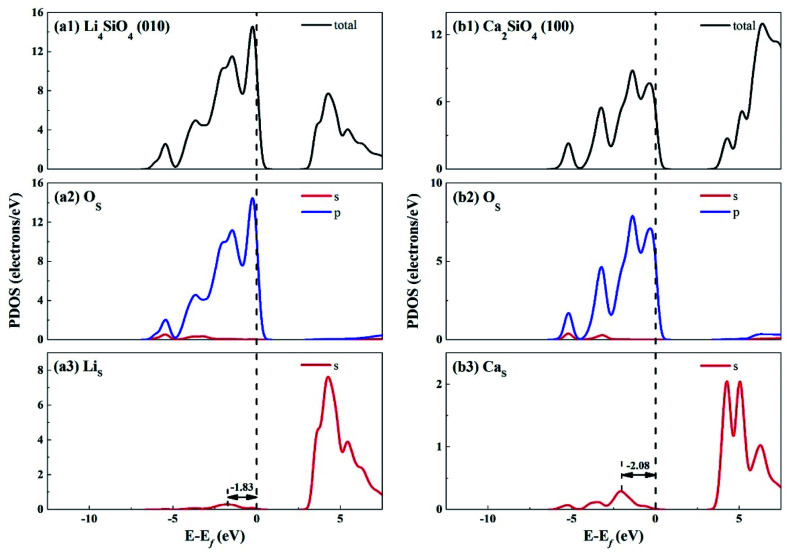
DOS analysis of the topmost surface layers of (a) Li_4_SiO_4_ (010) surface and (b) Ca_2_SiO_4_ (100) surface without CO_2_ adsorption. The black dashed line shows the Fermi level.

The electronic properties of the surfaces should differ from those of the bulks due to the dangling bonds or surface reconstruction. The electronic properties of Li_4_SiO_4_ (010) surface and Ca_2_SiO_4_ (100) surface were calculated as well. Fig. 6 shows the TDOS and PDOS of the topmost surface layers of Li_4_SiO_4_ (010) surface and Ca_2_SiO_4_ (100) surface. Although the surface TDOS resemble the bulk TDOS shown in Fig. 3 for both Li_4_SiO_4_ and Ca_2_SiO_4_, the states of O_S_ p orbitals are shifted up in energy, and the states in the region between −3 and 0 eV are about 79% of the total PDOS for O_S_ p orbitals for Li_4_SiO_4_ (010) surface, while that for Ca_2_SiO_4_ (100) surface is about 77%. Furthermore, p-band center of O_S_ atoms increased to −1.725 eV from −1.936 eV of O atoms in bulk Li_4_SiO_4_, and −1.939 eV from −2.103 eV in bulk Ca_2_SiO_4_. It can be deduced that the reactivity of the surface O_S_ atoms is enhanced compared with the O atoms in the bulks. Considering the states near to *E*_f_ and the p-band center levels, the O_S_ atoms in Li_4_SiO_4_ (010) surface are more prone to suffer the electrophilic attacks with respect to Ca_2_SiO_4_ (100) surface.

**Fig. 7:**
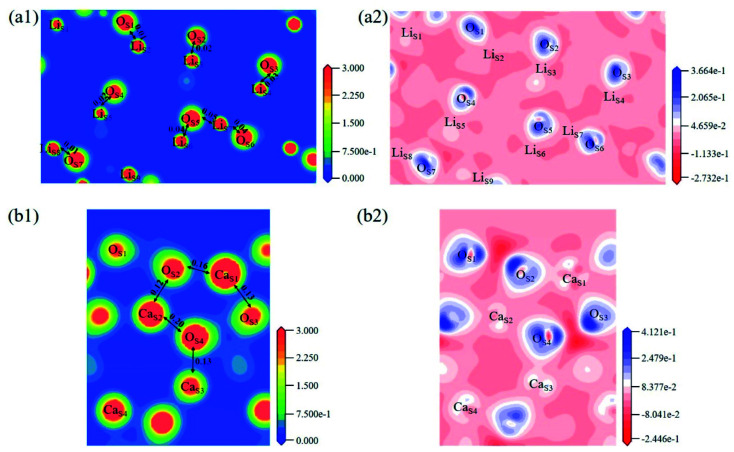
Contour maps of electron density distributions and differential charge density in the cross sections perpendicular to the (001) plane in (a) Li_4_SiO_4_ (010) surface and (b) Ca_2_SiO_4_ (100) surface.

The electron density and differential charge density of the topmost surface layer atoms are shown in Fig. 7. The covalent interaction between Li_S_ and O_S_ atoms is weaker than that in bulk, which can be seen from the charge population. While the covalent interaction between Ca_S_ and O_S_ atoms is stronger than that in bulk.

**Fig. 8:**
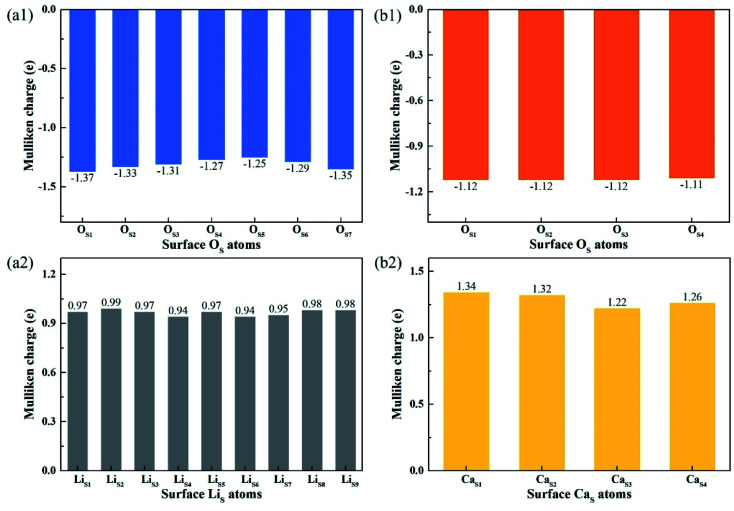
Mulliken charge analysis of atoms on the topmost surface layers of (a) Li_4_SiO_4_ (010) surface and (b) Ca_2_SiO_4_ (100) surface.

Fig. 8 shows the Mulliken charge of atoms on the topmost surface layer. There is a considerable difference of the charge between bulk atoms (listed in ESI†) and atoms on the topmost surface layer. The positive charge of surface Li_S_ and Ca_S_ atoms increases, and the negative charge of the O_S_ atoms increases compared with bulk atoms. And the deviation of Mulliken charge of surface Li_S_ atoms from the bulk atoms is relatively large, whereas the Mulliken charge of surface Ca_S_ and O_S_ atoms differ from their bulk atoms slightly. Furthermore, the O_S_ of Li_4_SiO_4_ (010) surface carry more negative charge than Ca_2_SiO_4_ (100) surface. According Lewis acid/base theory, O_S_ atoms of Li_4_SiO_4_ (010) surface are more basic and easier to lose electrons.

### 3.3. CO_2_ adsorption on the surfaces

The adsorption energy (*E*_ads_) of a CO_2_ molecule on Li_4_SiO_4_ (010) surface and Ca_2_SiO_4_ (100) surface is calculated according to the eqn (2):

2 *E*_ads_ = *E*_slab+CO_2__ − (*E*_slab_ + *E*_CO_2__)

where *E*_slab+CO_2__is the total energy of the surface with CO_2_ adsorption, *E*_CO_2__is the total energy of an isolated CO_2_ molecule. The lower adsorption energy describes the stronger binding between the adsorbed CO_2_ molecule and the surface, which reflects the stability of adsorption.

**Fig. 9:**
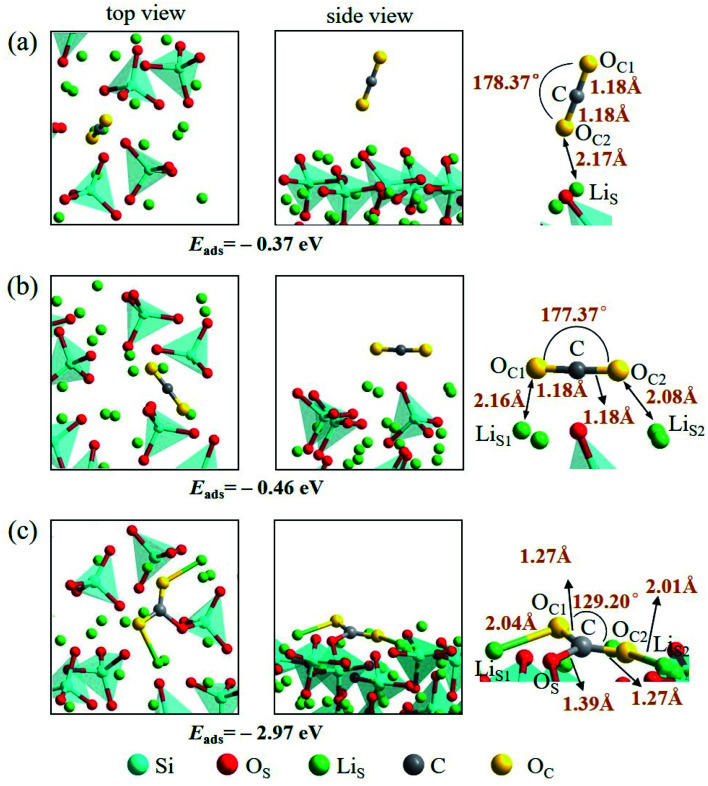
Three configurations of an adsorbed CO_2_ molecule on the Li_4_SiO_4_ (010) surface: (a) along the normal to the surface, (b) parallel to the surface, (c) bent configuration.

An isolated CO_2_ molecule has linear configuration with the length of C–O bond of 1.18 Å. Three adsorption configurations are presented in this study when a CO_2_ molecule is adsorbed on the Li_4_SiO_4_ (010) surface as shown in Fig. 9. In the first configuration, the adsorbed CO_2_ molecule almost remains linear configuration along the normal to the Li_4_SiO_4_ (010) surface. The distance between O atom in CO_2_ and the nearest Li_S_ atom is 2.17 Å, and adsorption energy is −0.37 eV. In the second configuration shown in Fig. 9b, the adsorbed CO_2_ molecule has a linear configuration parallel to the Li_4_SiO_4_ (010) surface, and the adsorption energy is −0.46 eV. In the third configuration shown in Fig. 9c, the adsorbed CO_2_ molecule is lying flat on the surface with the bent configuration. The C atom in CO_2_ forms a bond with a surface O_S_ atom with the O_S_–C distance of 1.39 Å, and its two oxygen atoms (O_C_) are coordinated to the two surface Li_S_ atoms, with the O_C_1__–Li_S_1__ and O_C_2__–Li_S_2__ distances are 2.04 and 2.01 Å, respectively. The adsorbed CO_2_ molecule is bent with an O_C_1__–C–O_C_2__ angle of 129.20° and the C–O_C_ length of 1.27 Å. The adsorption energy is −2.97 eV. The third bent configuration of the adsorbed CO_2_ on the Li_4_SiO_4_ (010) surface is energetically favorable over the other two configurations.

**Fig. 10:**
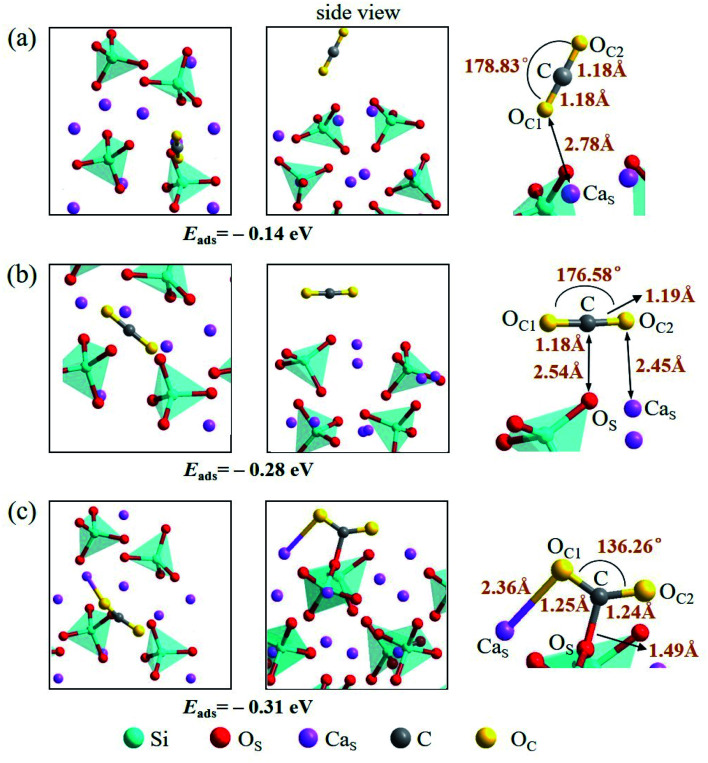
Three configurations of an adsorbed CO_2_ molecule on the Ca_2_SiO_4_ (100) surface: (a) along the normal to the surface, (b) parallel to the surface, (c) bent configuration.

There are also three adsorption configurations considered for an adsorbed CO_2_ molecule on the Ca_2_SiO_4_ (100) surface, as shown in Fig. 10. The first configuration, where an almost linearly CO_2_ is adsorbed, is in vertical orientation and tilted slightly to the Ca_2_SiO_4_ (100) surface, and the adsorption energy is −0.14 eV. The absorbed CO_2_ molecule is bent a little bit in the second configuration, which is in parallel orientation, and the adsorption energy is −0.28 eV. When the absorbed CO_2_ molecule lying on the Ca_2_SiO_4_ (100) surface in a bent configuration shown in Fig. 10c, the distance of C–O_S_ is 1.49 Å, and O_C_1__–C–O_C_2__ angle is 136.26°. The bent configuration has the lowest value of adsorption energy, −0.31 eV in the third configurations.

It is found that the bent configuration consisting of a CO_2_ molecule absorbed parallel along to the surface is the most thermodynamically stable among the three configurations considered here for both Li_4_SiO_4_ and Ca_2_SiO_4_ surfaces. Furthermore, the Li_4_SiO_4_ (010) surface has greater adsorption to CO_2_ than the Ca_2_SiO_4_ (100) surface due to the stronger bond between C atom in CO_2_ and the surface O_S_ atom for Li_4_SiO_4_.

### 3.4. Partial density of state analysis

**Fig. 11:**
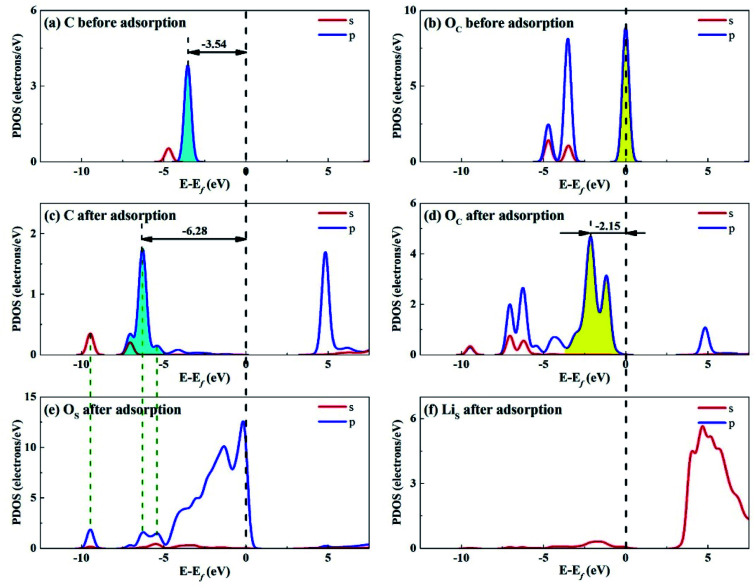
PDOS analysis of (a) C and (b) O_C_ atoms of CO_2_ before adsorption, (c) C and (d) O_C_ atoms of CO_2_ and (e) O_S_ and (f) Li_S_ atoms of Li_4_SiO_4_ (010) surface after adsorption.

**Fig. 12:**
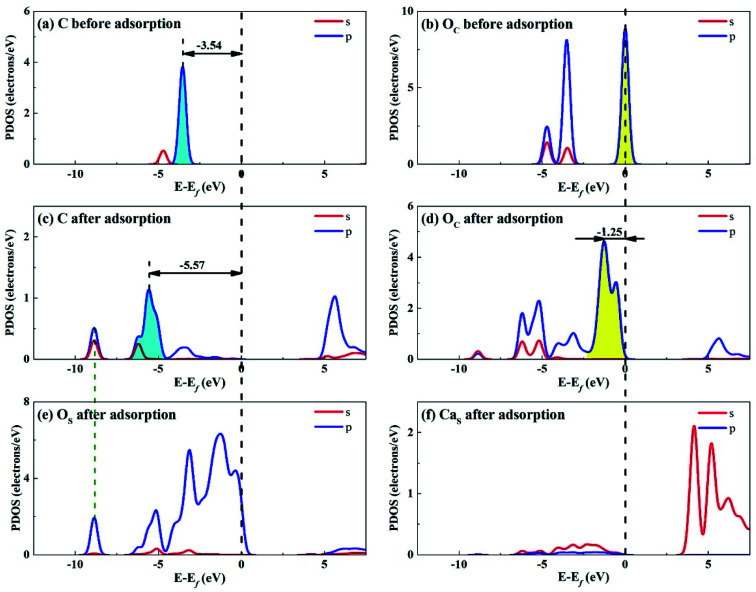
PDOS analysis of (a) C and (b) O_C_ atoms of CO_2_ before adsorption, (c) C and (d) O_C_ atoms of CO_2_ and (e) O_S_ and (f) Ca_S_ atoms of Ca_2_SiO_4_ (100) surface after adsorption.

Considering that the adsorption energy of adsorbed CO_2_ in bent configuration is the lowest, the PDOS calculations were only performed for this configuration, as shown in Fig. 11 and 12. It can be seen that the s and p orbitals of C and O_C_ of adsorbed CO_2_ molecule both move towards lower energy level and broaden compared with the isolated CO_2_ molecule, indicating that CO_2_ molecule becomes more stable after adsorption. C s and p orbitals of adsorbed CO_2_ are hybridized with O_S_ p orbitals, having bonding character between C and O_S_ atoms. The states in the region between −3 and 0 eV are about 72% for O_S_ p orbital for Li_4_SiO_4_ (010) surface after adsorption, which is decreased from 79% before adsorption, demonstrating that PDOS of O_S_ p orbitals is moved to lower energy level after CO_2_ adsorption. Furthermore, the PDOS peak of Li_S_ s orbital become weaker and broader after adsorption.

Fig. 12 shows the PDOS for CO_2_ and Ca_2_SiO_4_ (100) surface after adsorption. Similarly, the s and p orbitals of C and O_C_ of adsorbed CO_2_ move towards lower energy and broaden, but not as far as Li_4_SiO_4_ (010) surface. It can be deduced that CO_2_ adsorption on Li_4_SiO_4_ (010) surface is more stable. The states in the region between −3 and 0 eV are about 73% for O_S_ p orbitals for Ca_2_SiO_4_ (100) surface. The PDOS peaks for Ca_S_ s orbital become weaker and broader after adsorption, similar to Li_S_ s orbital.

**Fig. 13:**
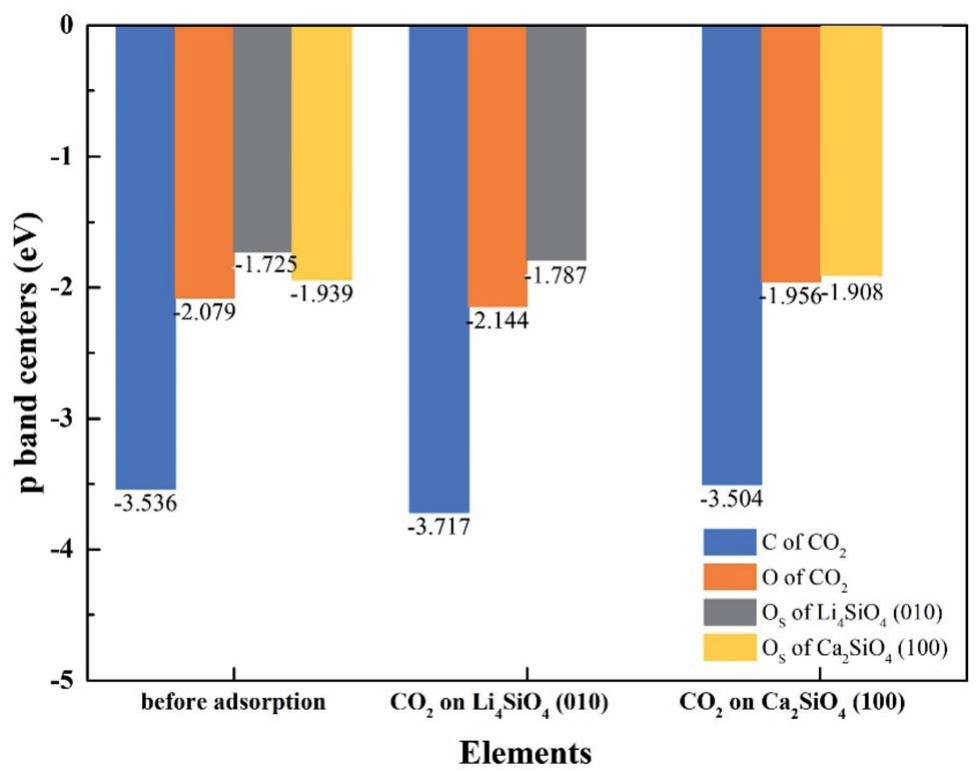
The p-band centers of C 2p and O 2p orbitals of CO_2_, Li_4_SiO_4_ (010) surface and Ca_2_SiO_4_ (100) surface before and after adsorption.

To better elucidate the different CO_2_ absorption behaviors of Li_4_SiO_4_ (010) and Ca_2_SiO_4_ (100) surfaces, the p-band centers of C and O_C_ in CO_2_ and O_S_ on the surfaces with and without CO_2_ absorption were calculated, and the results were shown in Fig. 13. Comparing the p-band centers of O_S_ atoms on two clean surfaces, it can be found that the p-band center of O_S_ atoms on the Li_4_SiO_4_ (010) surface is closer to the *E*_f_ than that of Ca_2_SiO_4_ (100) surface, which means the O_S_ atoms of Li_4_SiO_4_ (010) surface are more active and easier to transfer electrons to CO_2_ absorbed. When CO_2_ is adsorbed on the Li_4_SiO_4_ (010) surface, it is obvious that p-band centers of C, O_C_ and O_S_ atoms are farther away from the *E*_f_, indicating that CO_2_ absorbed and O_S_ become stable. On the other hand, energy-down shift of the p-band centers of C, O_C_ and O_S_ atoms of Ca_2_SiO_4_ (100) surface due to CO_2_ adsorption is much smaller compared with Li_4_SiO_4_ (010) surface, probably leading to its higher absorption energy and less CO_2_ absorption.

### 3.5. Mulliken charge analysis

**Fig. 14:**
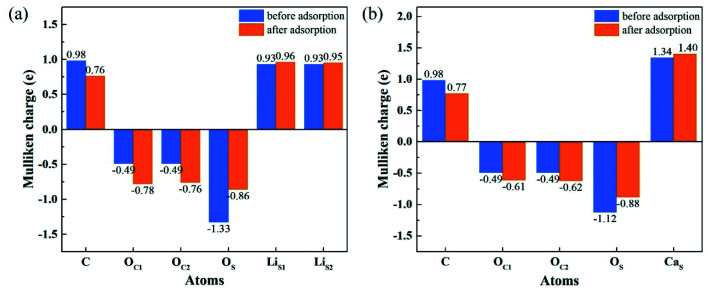
Mulliken charge analysis of adsorbed CO_2_ molecule and (a) Li_4_SiO_4_ (010) surface, and (b) Ca_2_SiO_4_ (100) surface before and after adsorption.

To understand the interactions and charge distributions associated with CO_2_ adsorbed with the most energetically favorable configurations on Li_4_SiO_4_ (010) and Ca_2_SiO_4_ (100) surfaces, a Mulliken charge anlysis was performed. The detail date of Mulliken charges for CO_2_ and Li_4_SiO_4_ (010) and Ca_2_SiO_4_ (100) surfaces before and after adsorption were shown in Fig. 14. For CO_2_ adsorbed on Li_4_SiO_4_ (010) surface, it is found that the Mulliken charge on a surface O_S_ atom changes from −1.33*e* to −0.86*e*, while the charges on the surface Li_S_1__ and Li_S_2__ atoms increase from 0.93*e* to 0.96*e*, and from 0.93*e* to 0.95*e*, respectively. It can be deduced that CO_2_ adsorption induces the net electron loss of Li_4_SiO_4_ (010) surface. On the other side, the Mulliken charge of C atom in CO_2_ adsorbed decreases from 0.98*e* to 0.76*e*, and the charges of O_C_1__ and O_C_2__ from −0.49*e* to −0.78*e* and −0.76*e*, respectively, which means that CO_2_ adsorbed gains the charges from Li_4_SiO_4_ (010) surface.

In the case of CO_2_ adsorption on Ca_2_SiO_4_ (100) surface, it is found that charge gain for C atom in CO_2_ adsorbed is similar to that on Li_4_SiO_4_ (010) surface, but charges gained by O_C1_ and O_C2_ atoms are fewer. Furthermore, the Mulliken charge on an O_S_ atom on Ca_2_SiO_4_ (100) surface changes from −1.12*e* to −0.88*e*, and the charge on the surface Ca_S_ from 1.34*e* to 1.40*e*. The net charge transfer from Ca_2_SiO_4_ (100) surface to CO_2_ adsorbed is much less compared to that Li_4_SiO_4_ (010) surface.

The charge population between C in CO_2_ adsorbed and a surface O_S_ atom was calculated to be 0.62 and 0.45 for Li_4_SiO_4_ (010) and Ca_2_SiO_4_ (100) surfaces, respectively. It can be deduced that the C–O_S_ covalent interaction on Li_4_SiO_4_ (010) surface is even stronger, which leads to the stronger adsorption of CO_2_.

## 4. Conclusions

A density functional theory calculation was conducted to research the CO_2_ adsorption on the Li_4_SiO_4_ (010) and Ca_2_SiO_4_ (100) surfaces. The bent configuration consisting of a CO_2_ molecule adsorbed parallel along to the surface is the most thermodynamically favorable for Li_4_SiO_4_ and Ca_2_SiO_4_ surfaces. And the adsorption energy of Li_4_SiO_4_ (010) surface is −2.97 eV, more negative than Ca_2_SiO_4_ (100) surface, −0.31 eV. Li_4_SiO_4_ (010) surface is more favorable for forming a stronger covalent bond between a surface O_S_ atom to the C atom of CO_2_ adsorbed and transferring more charges to adsorbed CO_2_. In addition, it was found that the Mulliken charge of O_S_ atoms on the Li_4_SiO_4_ (010) is more negative, and its p-band center is closer to the E_f_, which implies O_S_ atoms of Li_4_SiO_4_ (010) are more active and more likely serve as the electron donors with respect to Ca_2_SiO_4_ (100) surface.

## Conflicts of interest

There are no conflicts to declare.

## Supplementary Material

RA-012-D2RA01021F-s001

RA-012-D2RA01021F-s002

RA-012-D2RA01021F-s003

## References

[cit1] Ben-Mansour R., Habib M. A., Bamidele O. E., Basha M., Qasem N. A. A., Peedikakkal A., Laoui T., Ali M. (2016). Appl. Energy.

[cit2] Seggiani M., Puccini M., Vitolo S. (2013). Int. J. Greenhouse Gas Control.

[cit3] Kato M., Yoshikawa S., Nakagawa K. (2002). J. Mater. Sci. Lett..

[cit4] Kato M., Nakagawa K., Essaki K., Maezawa Y., Takeda S., Kogo R., Hagiwara Y. (2005). Int. J. Appl. Ceram. Technol..

[cit5] Wang K., Yin Z., Zhao P. (2016). Ceram. Int..

[cit6] Bretado M. E., Velderrain V. G., Gutiérrez D. L., Collins-Martínez V., Ortiz A. L. (2005). Catal. Today.

[cit7] Essaki K., Kato M. (2005). J. Mater. Sci..

[cit8] Rodríguez-Mosqueda R., Pfeiffer H. (2010). J. Phys. Chem..

[cit9] Venegas M. J., Fregoso-Israel E., Escamilla R., Pfeiffer H. (2007). Ind. Eng. Chem. Res..

[cit10] Jeoung S., Lee J. H., Kim H. Y., Moon H. R. (2016). Thermochim. Acta.

[cit11] Romero-Ibarra I. C., Ortiz-Landeros J., Pfeiffer H. (2013). Thermochim. Acta.

[cit12] Mejía-Trejo V. L., Fregoso-Israel E., Pfeiffer H. (2008). Chem. Mater..

[cit13] Tomarov G. V., Petrov Y. V., Shipkov A. A., Dovgii O. A., Semenov V. N., Mikhailov A. V. (2008). Therm. Eng..

[cit14] Zhao M., Shi J., Zhong X., Tian S., Blamey J., Jiang J., Fennell P. S. (2014). Energy Environ. Sci..

[cit15] Zhao M., Song Y., Ji G., Zhao X. (2018). Energy Fuels.

[cit16] Kim Y. S., Kang S. G. (2019). Appl. Surf. Sci..

[cit17] Kumar A., Ropital F., de Bruin T., Diawara B. (2020). Appl. Surf. Sci..

[cit18] Kang S. G. (2020). J. CO_2_ Util..

[cit19] Duan Y., Parlinski K. (2011). Phys. Rev. B: Condens. Matter Mater. Phys..

[cit20] Tang T., Chen P., Luo W., Luo D., Wang Y. (2012). J. Nucl. Mater..

[cit21] Kong X., Yu Y., Ma S., Gao T., Xiao C., Chen X. (2018). Chem. Phys. Lett..

[cit22] Qi C., Spagnoli D., Fourie A. (2020). Appl. Surf. Sci..

[cit23] Wang Q., Manzano H., López-Arbeloa I., Shen X. (2018). Minerals.

[cit24] Wang Q., Li F., Shen X., Shi W., Li X., Guo Y., Xiong S., Zhu Q. (2014). Cem. Concr. Res..

[cit25] Payne M. C., Teter M. P., Allan D. C., Arias T. A., Joannopoulos J. D. (1992). Rev. Mod. Phys..

[cit26] Perdew J. P., Burke K., Ernzerhof M. (1996). Phys. Rev. Lett..

[cit27] Hammer B. (1999). Phys. Rev. B: Condens. Matter Mater. Phys..

[cit28] Ramalho J. P. P., Gomes J. R. B., Illas F. (2013). RSC Adv..

[cit29] Grimme S., Antony J., Ehrlich S., Krieg H. (2010). J. Chem. Phys..

[cit30] Chadi D. J. (1977). Phys. Rev. B.

[cit31] Pfrommer B. G., Côté M., Louie S. G., Cohen M. L. (1997). J. Comput. Phys..

[cit32] Belsky A., Hellenbrandt M., Karena V. L., Lukschb P. (2002). Acta Crystallogr..

[cit33] Zhang R., Ma S., Wang Q., Xiao C., Zhang C., Gao T. (2020). Ceram. Int..

[cit34] Guan Q., Gao T., Shen Y., Ma S., Lu T., Chen X., Xiao C., Long X. (2015). Int. J. Mod. Phys. B.

[cit35] Tranqui D., Shannont R. D., Chen H. Y. (1979). Acta Crystallogr. B.

[cit36] Haitao C., Xuefei H., Weigang H. (2018). Rare Met. Mater. Eng..

[cit37] Chan C.-J., Kriven W. M., Young J. F. (1988). J. Am. Ceram. Soc..

[cit38] Kim Y. J., Nettleship I., Kriven W. M. (1992). J. Am. Ceram. Soc..

[cit39] Marenich A. V., Jerome S. V., Cramer C. J., Truhlar D. G. (2012). J. Chem. Theory Comput..

[cit40] Jost K. H., Ziemer B., Seydel R. (1977). Acta Crystallogr..

[cit41] Tao W., Zhu C., Xu Q., Li S., Xiong X., Cheng H., Zou X., Lu X. (2020). ACS Omega.

[cit42] Durgun E., Manzano H., Kumar P. V., Grossman J. C. (2014). J. Phys. Chem. C.

[cit43] Zhang W.-B., Chen C., Zhang S.-Y. (2013). J. Phys. Chem. C.

