# Supplementary material for: Revealing the different performance of Li4SiO4 and Ca2SiO4 for CO2 adsorption by density functional theory
Source: RSC Adv. 2022 Apr 11;12(18):11190–201. doi: 10.1039/d2ra01021f (PMC8996757; doi:10.1039/d2ra01021f)
Supplement: RA-012-D2RA01021F-s003 [file RA-012-D2RA01021F-s003.pdf]

Electronic Supplementary Material (ESI) for RSC Advances.
This journal is © The Royal Society of Chemistry 2022

Supporting Information:

Revealing the different performance of Li_4SiO_4 and Ca_2SiO_4 for CO_2 adsorption by density functional theory

Wenjing Yu[a], Qian Xu*[a], Shenggang Li*[b], Xiaolu Xiong[a], Hongwei Cheng[a], Xingli Zou[a] and Xionggang Lu[a]

a The State Key Laboratory of Advanced Special Steel, Shanghai Key Laboratory of Advanced Ferrometallurgy, School of Materials Science and Engineering, Shanghai University, Shanghai, P. R. China

b CAS Key Laboratory of Low-Carbon Conversion Science and Engineering, Shanghai Advanced Research Institute, Chinese Academy of Sciences, Shanghai, P. R. China

Corresponding author. E-mail: qianxu@shu.edu.cn (Qian Xu); lisg@sari.ac.cn (S. Li)

Table S1 Mulliken charge analysis of Li atoms in Li_4SiO_4.

Atom	Mulliken charge	Atom	Mulliken charge	Atom	Mulliken charge	Atom	Mulliken charge
Li1	0.84	Li15	0.86	Li29	0.84	Li43	0.84
Li2	0.84	Li16	0.88	Li30	0.84	Li44	0.80
Li3	0.84	Li17	0.87	Li31	0.84	Li45	0.79
Li4	0.84	Li18	0.85	Li32	0.87	Li46	0.80
Li5	0.87	Li19	0.84	Li33	0.86	Li47	0.85
Li6	0.86	Li20	0.84	Li34	0.88	Li48	0.84
Li7	0.88	Li21	0.84	Li35	0.87	Li49	0.86
Li8	0.87	Li22	0.84	Li36	0.85	Li50	0.88
Li9	0.85	Li23	0.87	Li37	0.85	Li51	0.84
Li10	0.84	Li24	0.86	Li38	0.84	Li52	0.83
Li11	0.84	Li25	0.88	Li39	0.86	Li53	0.84
Li12	0.84	Li26	0.87	Li40	0.88	Li54	0.80
Li13	0.84	Li27	0.85	Li41	0.84	Li55	0.79
Li14	0.87	Li28	0.84	Li42	0.83	Li56	0.80

Table S2 Mulliken charge analysis of O atoms in Li_4SiO_4.

Atom	Mulliken charge	Atom	Mulliken charge	Atom	Mulliken charge	Atom	Mulliken charge
O1	–1.27	O15	–1.27	O29	–1.29	O43	–1.29
O2	–1.27	O16	–1.27	O30	–1.25	O44	–1.25
O3	–1.28	O17	–1.28	O31	–1.29	O45	–1.29
O4	–1.27	O18	–1.27	O32	–1.29	O46	–1.29
O5	–1.26	O19	–1.26	O33	–1.26	O47	–1.26

O6	–1.27	O20	–1.27	O34	–1.24	O48	–1.24
O7	–1.29	O21	–1.29	O35	–1.27	O49	–1.27
O8	–1.27	O22	–1.27	O36	–1.28	O50	–1.28
O9	–1.27	O23	–1.27	O37	–1.28	O51	–1.28
O10	–1.28	O24	–1.28	O38	–1.27	O52	–1.27
O11	–1.27	O25	–1.27	O39	–1.29	O53	–1.29
O12	–1.26	O26	–1.26	O40	–1.27	O54	–1.27
O13	–1.27	O27	–1.27	O41	–1.28	O55	–1.28
O14	–1.29	O28	–1.29	O42	–1.28	O56	–1.28

Table S3 Mulliken charge analysis of Ca and O atoms in Ca_2SiO_4.

Atom	Mulliken charge	Atom	Mulliken charge	Atom	Mulliken charge	Atom	Mulliken charge
Ca1	1.27	Ca7	1.27	O5	–1.09	O11	–1.10
Ca2	1.26	Ca8	1.26	O6	–1.10	O12	–1.10
Ca3	1.27	O1	–1.09	O7	–1.10	O13	–1.09
Ca4	1.26	O2	–1.10	O8	–1.10	O14	–1.10
Ca5	1.27	O3	–1.10	O9	–1.09	O15	–1.10
Ca6	1.26	O4	–1.10	O10	–1.10	O16	–1.10

Considering that the adsorption energy of adsorbed CO_2 in bent configuration is the lowest, the vibration frequencies were only calculated for this configuration, as displayed in Table S4. The vibrational frequencies of CO_2 adsorbed on Li_4SiO_4 (010) and Ca_2SiO_4 (100) surfaces shifted significantly from those of isolated CO_2 molecule. Fig.S1 shows the corresponding vibrational model of adsorbed CO_2.

Table S4 Vibrational frequencies (cm^{-1}) of isolated and adsorbed CO_2 in bent configuration.

Configuration	Asymmetric stretch	Symmetric stretch	Bending vibration	$C\text{-}O_S$
Isolated CO_2	2365	1334	646	
adsorbed CO_2 on Li_4SiO_4 (010)	1568	1254	764	1006
adsorbed CO_2 on Ca_2SiO_4 (100)	1679	1221	738	827

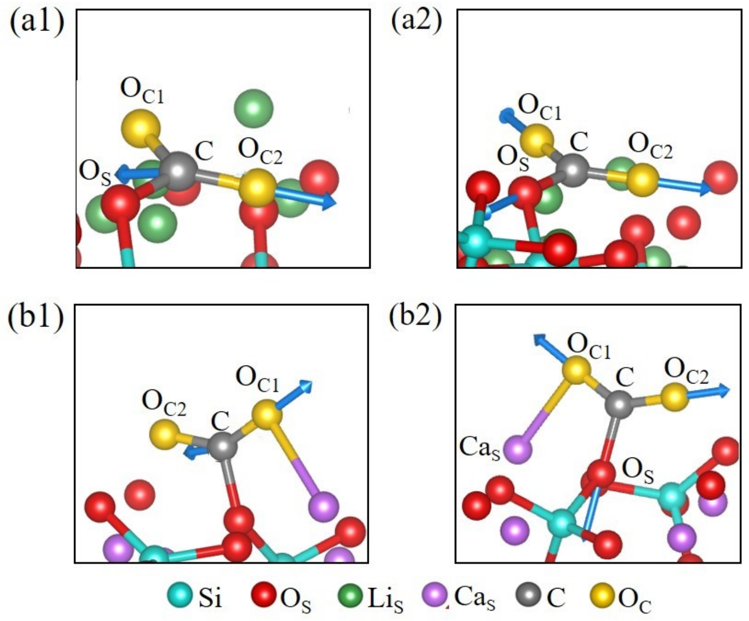

Fig. S1. Vibrational modes of adsorbed CO_2 molecule: (a1) asymmetric stretching, (a2) symmetric stretching on Li_4SiO_4 (010) surface and (b1) asymmetric stretching, (b2) symmetric stretching on Ca_2SiO_4 (100) surface.

Table S5 The fractional coordinates of atoms in Li_4SiO_4.

Atom	Fractional coordinates		
	u	v	w
Li1	0.382673	0.002866	0.408360
Li2	0.811847	0.002090	0.545327
Li3	0.663326	0.006108	0.826900
Li4	0.522364	1.004921	0.117369
Li5	0.191649	0.969313	0.014491
Li6	0.333241	0.964591	0.732463
Li7	0.949715	0.031048	0.707157
Li8	0.079250	0.014688	0.866891
Li9	0.237800	0.038150	0.597954
Li10	-0.382670	0.502866	-0.408360
Li11	-0.811850	0.502090	-0.545330
Li12	-0.663330	0.506108	-0.826900
Li13	-0.522360	1.504921	-0.117370
Li14	-0.191650	1.469313	-0.014490
Li15	-0.333240	1.464591	-0.732460
Li16	-0.949720	0.531048	-0.707160
Li17	-0.079250	0.514688	-0.866890
Li18	-0.237800	0.538150	-0.597950
Li19	-0.382670	-0.002870	-0.408360
Li20	-0.811850	-0.002090	-0.545330
Li21	-0.663330	-0.006110	-0.826900

Li22	-0.522360	-1.004920	-0.117370
Li23	-0.191650	-0.969310	-0.014490
Li24	-0.333240	-0.964590	-0.732460
Li25	-0.949720	-0.031050	-0.707160
Li26	-0.079250	-0.014690	-0.866890
Li27	-0.237800	-0.038150	-0.597950
Li28	0.382673	0.497134	0.408360
Li29	0.811847	0.497910	0.545327
Li30	0.663326	0.493892	0.826900
Li31	0.522364	-0.504920	0.117369
Li32	0.191649	-0.469310	0.014491
Li33	0.333241	-0.464590	0.732463
Li34	0.949715	0.468952	0.707157
Li35	0.079250	0.485312	0.866891
Li36	0.237800	0.461850	0.597954
Li37	0.273809	0.250000	0.859186
Li38	0.418333	0.250000	0.548899
Li39	0.127996	0.250000	0.145984
Li40	0.971183	0.250000	0.428925
Li41	0.452137	0.250000	0.276875
Li42	0.728182	0.250000	0.695152
Li43	0.589839	0.250000	0.983003
Li44	0.141723	0.250000	0.731525
Li45	0.006133	0.250000	0.010710
Li46	0.878098	0.250000	0.286783
Li47	-0.273810	0.750000	-0.859190
Li48	-0.418330	0.750000	-0.548900
Li49	-0.128000	0.750000	-0.145980
Li50	-0.971180	0.750000	-0.428930
Li51	-0.452140	0.750000	-0.276880
Li52	-0.728180	0.750000	-0.695150
Li53	-0.589840	0.750000	-0.983000
Li54	-0.141720	0.750000	-0.731530
Li55	-0.006130	0.750000	-0.010710
Li56	-0.878100	0.750000	-0.286780
O1	0.356709	0.024866	0.060465
O2	0.785475	0.030107	0.196306
O3	0.498121	0.026844	0.770084
O4	0.216536	0.023722	0.346294
O5	0.068813	0.030602	0.637933
O6	0.927391	0.030932	0.916095
O7	0.654005	0.026732	0.481683
O8	-0.356710	0.524866	-0.060470
O9	-0.785480	0.530107	-0.196310

O10	-0.498120	0.526844	-0.770080
O11	-0.216540	0.523722	-0.346290
O12	-0.068810	0.530602	-0.637930
O13	-0.927390	0.530932	-0.916100
O14	-0.654010	0.526732	-0.481680
O15	-0.356710	-0.024870	-0.060470
O16	-0.785480	-0.030110	-0.196310
O17	-0.498120	-0.026840	-0.770080
O18	-0.216540	-0.023720	-0.346290
O19	-0.068810	-0.030600	-0.637930
O20	-0.927390	-0.030930	-0.916100
O21	-0.654010	-0.026730	-0.481680
O22	0.356709	0.475134	0.060465
O23	0.785475	0.469893	0.196306
O24	0.498121	0.473156	0.770084
O25	0.216536	0.476278	0.346294
O26	0.068813	0.469398	0.637933
O27	0.927391	0.469068	0.916095
O28	0.654005	0.473268	0.481683
O29	0.198009	0.250000	0.959462
O30	0.614786	0.250000	0.097918
O31	0.331704	0.250000	0.676042
O32	0.059475	0.250000	0.245620
O33	0.906508	0.250000	0.528084
O34	0.760242	0.250000	0.811563
O35	0.474002	0.250000	0.394183
O36	0.420824	0.250000	0.933138
O37	0.844772	0.250000	0.070550
O38	0.555612	0.250000	0.640212
O39	0.281305	0.250000	0.220026
O40	0.132789	0.250000	0.513400
O41	0.992621	0.250000	0.792628
O42	0.695697	0.250000	0.352749
O43	-0.198010	0.750000	-0.959460
O44	-0.614790	0.750000	-0.097920
O45	-0.331700	0.750000	-0.676040
O46	-0.059480	0.750000	-0.245620
O47	-0.906510	0.750000	-0.528080
O48	-0.760240	0.750000	-0.811560
O49	-0.474000	0.750000	-0.394180
O50	-0.420820	0.750000	-0.933140
O51	-0.844770	0.750000	-0.070550
O52	-0.555610	0.750000	-0.640210
O53	-0.281310	0.750000	-0.220030

O54	-0.132790	0.750000	-0.51340
O55	-0.992620	0.750000	-0.792630
O56	-0.695700	0.750000	-0.352750
Si1	0.340420	0.250000	0.005836
Si2	0.751724	0.250000	0.137589
Si3	0.476914	0.250000	0.713706
Si4	0.200632	0.250000	0.292318
Si5	0.039110	0.250000	0.578038
Si6	0.894808	0.250000	0.856011
Si7	0.614530	0.250000	0.428549
Si8	-0.340420	0.750000	-0.005840
Si9	-0.751720	0.750000	-0.137590
Si10	-0.476910	0.750000	-0.713710
Si11	-0.200630	0.750000	-0.292320
Si12	-0.039110	0.750000	-0.578040
Si13	-0.894810	0.750000	-0.856010
Si14	-0.614530	0.750000	-0.428550

Table S6 The fractional coordinates of atoms in Ca_2SiO_4.

Atom	Fractional coordinates		
	u	v	w
Ca1	0.228483	0.339181	0.429580
Ca2	0.224207	-0.003760	0.703924
Ca3	0.271517	0.839181	0.070420
Ca4	0.275793	0.496241	-0.203920
Ca5	-0.228480	-0.339180	-0.429580
Ca6	-0.224210	0.003759	-0.703920
Ca7	0.728483	0.160819	0.929580
Ca8	0.724207	0.503759	1.203924
O1	0.219663	0.011419	0.444317
O2	0.014682	0.667842	0.364502
O3	0.475601	0.750073	0.305485
O4	0.350156	0.670661	0.572730
O5	0.280337	0.511419	0.055683
O6	0.485318	1.167842	0.135498
O7	0.024399	1.250073	0.194515
O8	0.149844	1.170661	-0.072730
O9	-0.219660	-0.011420	-0.444317
O10	-0.014680	-0.667840	-0.364502
O11	-0.475600	-0.750070	-0.305485
O12	-0.350160	-0.670660	-0.572730
O13	0.719663	0.488581	0.944317
O14	0.514682	-0.167840	0.864502
O15	0.975601	-0.250070	0.805485

O16	0.850156	-0.170660	1.072730
Si1	0.267837	0.780610	0.419122
Si2	0.232163	1.280610	0.080878
Si3	-0.267840	-0.780610	-0.419120
Si4	0.767837	-0.280610	0.919122
